# The Role of pH_i_ in Intestinal Epithelial Proliferation–Transport Mechanisms, Regulatory Pathways, and Consequences

**DOI:** 10.3389/fcell.2021.618135

**Published:** 2021-01-22

**Authors:** Mahdi Amiri, Ursula E. Seidler, Katerina Nikolovska

**Affiliations:** Department of Gastroenterology, Hannover Medical School, Hannover, Germany

**Keywords:** intracellular pH, epithelial ion transport, proliferation, signaling pathways, intestinal epithelial cells

## Abstract

During the maturation of intestinal epithelial cells along the crypt/surface axis, a multitude of acid/base transporters are differentially expressed in their apical and basolateral membranes, enabling processes of electrolyte, macromolecule, nutrient, acid/base and fluid secretion, and absorption. An intracellular pH (pH_i_)-gradient is generated along the epithelial crypt/surface axis, either as a consequence of the sum of the ion transport activities or as a distinctly regulated entity. While the role of pH_i_ on proliferation, migration, and tumorigenesis has been explored in cancer cells for some time, emerging evidence suggests an important role of the pH_i_ in the intestinal stem cells (ISCs) proliferative rate under physiological conditions. The present review highlights the current state of knowledge about the potential regulatory role of pH_i_ on intestinal proliferation and differentiation.

## Introduction

Mechanisms of acid/base control in the gastrointestinal tract came into focus a century ago, because during the decades of very high gastric ulcer prevalence, a relationship between peptic ulcers and gastric acidity had been recognized (Banic et al., 2011). The ability to assess pH_i_ in mammalian gastrointestinal cells with the use of fluorescent dyes (Thomas, 1986) made the study of pH_i_-recovery after an acidic or alkaline insult possible, as well as the delineation of the involved ion transporters (Flemstrom and Isenberg, 2001; Kaunitz and Akiba, 2006; Seidler, 2013). pH_i_ measurements have also been utilized to outline the transport proteins involved in intestinal absorptive and secretory processes (Zachos et al., 2005; Hug et al., 2011; Seidler and Nikolovska, 2019).

**FIGURE 1:**
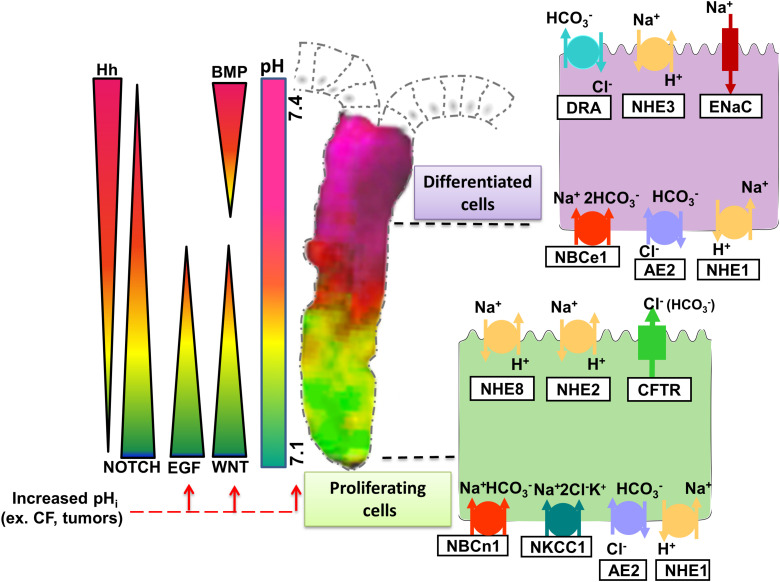
Representation of experimentally determined pH_i_ gradient and the major signaling cascades in the colonic crypt that together control intestinal homeostasis. Wnt and EGF are essential for proliferation and maintenance of ISCs. Notch signaling targets the proliferating cells directing them to secretory or absorptive lineage differentiation. Hedgehog (Hh), expressed by epithelial cells in the upper part of the crypt maintains the myofibroblasts and prompts BMP ligand expression, which then promotes differentiation while restraining cell proliferation of the epithelial cells. Since they are involved in different steps of intestinal homeostasis, the signaling pathways form an opposing gradient along the crypt axis. The activity of the signaling pathways is influenced by a pH_i_ gradient spread along the crypt, with more acidic pH_i_ values present at the bottom and more alkaline values toward the cryptal surface. The pH_i_ gradient is generated by the activity of the different ion transporters expressed on the intestinal epithelial cells. Proliferating cells are characterized by high activity/expression of CFTR, NHE2, and NHE8 on their apical membrane, and NBCn1, NKCC1, AE2, and NHE1 on the basolateral membrane. As the cells transition from proliferating to differentiated state, the expression of NHE3, DRA (Slc26a3), and ENaC becomes more dominant at the apical membrane and NBCe1, NHE1, and AE2 on the basolateral membrane. Tightly regulated expression and activity of the ion transporters at different segments of the crypt allows maintenance of distinct pH_i_ values that in turn control the activity of the signaling pathways essential for synchronized proliferation and differentiation to retain intestinal homeostasis. Increase of pH_i_ caused by alterations in ion transporter expression/activity (e.g., Cystic Fibrosis-CF) or tumors leads to activation of certain signaling pathways and hyperproliferation.

In the apical membranes, the anion channel cystic fibrosis transmembrane conductance regulator (CFTR) and the Cl^–^/HCO_3_^–^ exchanger SLC26A3 (and possibly SLC26A6) export HCO_3_^–^ into the lumen and are therefore “acid loaders.” Likewise, proton-coupled nutrient transporters load the enterocytes with acid moieties during digestion. The activity of acid loaders is counteracted by the apical acid extruders, the Na^+^/H^+^ exchangers NHE2, NHE3, and NHE8 (SLC9A2/3/8); a process that results in salt and water absorption. Apical proton ATPases are also expressed in various cell types along the GI tract. The basolateral membranes also express both acid loaders, such as the Cl^–^/HCO_3_^–^ exchanger AE2 (SLC4A2), and acid extruders, such as the NHE1 and the Na^+^/HCO_3_^–^ cotransporters NBCn1 (SLC4A7) and NBCe1 (SLC4A4). Immunostaining and *in situ* hybridization techniques localized these transporters in the respective membranes, often with an expression gradient along cryptal or crypt/villus axis, and along the proximal to distal gut axis (Strong et al., 1994; Ameen et al., 1995; Ameen N. et al., 2000; Ameen N.A. et al., 2000; Alper et al., 1999; Chu et al., 2002; Jacob et al., 2002; Wang et al., 2002; Boedtkjer et al., 2008; Jakab et al., 2011; Singh et al., 2013b). Figure 1 depicts a colonic crypt, with experimentally determined pH_i_-gradient along its axis, and the relevant acid/base transporters on the apical and basolateral membranes.

The role of the steady-state pH_i_ in the cellular physiology of the intestinal epithelium has not been addressed experimentally, partially because of experimental uncertainties in the calibration process that allows conversion of the fluorometric intensity into an actual pH_i_ value (O’Connor and Silver, 2013), and because of the short lifetime of isolated intestinal mucosal preparations. Recent progress toward preservation of functional intestinal stem cells and therefore cultivation of native intestinal epithelium in so-called “organoid cultures” has enabled scientists to observe cellular functions, including the pH_i_, of the intestinal epithelium in “steady-state.” This minireview highlights recent novel findings regarding the role of pH_i_ in intestinal proliferation and discusses the potential role of pH_i_ in the signaling pathways that regulate the constant renewal of the intestinal mucosa.

### pH_i_-Regulatory Studies in the Intestinal Epithelium

Temporal changes in the pH_i_ of epithelial cells in the GI tract are imposed physiologically due to changes in luminal pH. For example, the consequences of short-term exposure of the mouse gastric and duodenal epithelium to acidic luminal perfusate (mimicking the stage of gastric emptying), or of the colonic epithelium by short chain fatty acids (mimicking bacterial metabolism), have been studied in detail. The epithelium counteracts this intracellular acidification by activation and/or rapid trafficking of a variety of ion transporters to the brush border and basolateral membrane, facilitating proton extrusion, and HCO_3_^–^ import to re-establish the resting pH_i_ (Chu and Montrose, 1995; Akiba et al., 2001; Singh et al., 2013a). These processes are coordinated by a large array of neural, paracrine, and direct epithelial regulatory mechanisms (Smith et al., 2006; Singh et al., 2012; Takeuchi et al., 2012; Akiba and Kaunitz, 2014).

In contrast to transient pH_i_ alterations, the consequences of sustained deviations from the resting pH_i_ as a result of impaired ion transport have hardly been studied in the native intestinal epithelium. For decades, the role of steady-state pH_i_ alterations has been addressed primarily in tumor cells. In cancer cells numerous H^+^ extrusion and base loading mechanisms are upregulated, which generally leads to an inverted transmembrane pH gradient, characterized by alkalization of intracellular pH and extracellular acidosis, which is considered a hallmark of cancer metabolism (Webb et al., 2011; Swietach et al., 2014; Pedersen et al., 2017; Flinck et al., 2018; Becker and Deitmer, 2020; Liu et al., 2020). In this scenario, both the high intracellular and the low extracellular pH contribute to the malignant behavior (Pillai et al., 2019; Boedtkjer and Pedersen, 2020). Early studies supporting a role of mitogenic activation of Na^+^/H^+^ exchange and intracellular alkalinization in proliferation of non-transformed cells, such as fibroblasts (Grinstein et al., 1989) has been met with criticism because a concomitant activation of acid loaders abolished the rise in pH_i_ in fibroblasts in the presence of CO_2_/HCO_3_^–^ in the medium (Gillies and Martinezzaguilan, 1991). Recent technological advances and an expanded knowledge about the molecular nature of acid/base transporters as well as the mechanisms of epithelial growth and differentiation allow addressing the question about the influence of pH_i_ in epithelial homeostasis.

### Alkaline Steady-State pH_i_ Caused by Loss of the CFTR Channel in Intestinal Stem Cells (ISCs) Is a Causative Factor in ISC Hyperproliferation

Simpson et al. (2005) had identified an alkaline pH_i_ in the intact epithelium of *Cftr^–/–^* compared to identically treated wild type (*wt*) mouse duodenal mucosa. When techniques became available for intestinal stem cell maintenance and thus long term culture of native intestinal organoids, the same group used the technique to demonstrate a sustained alkalized resting pH_i_ in the epithelial cells of small intestinal organoids grown from *Cftr^–/–^* crypts (Walker et al., 2016). Interestingly, the group demonstrated that the alkaline pH_i_ was not primarily due to the defective HCO_3_*^–^* conductance via CFTR, but to its defective Cl*^–^* conductance, resulting in intracellular Cl*^–^* retention and an inability of the basolateral acid loader AE2 to export HCO_3_*^–^*_*i*_ in exchange for Cl*^–^*_*o*_. Accordingly, pH_i_ could be normalized by normalizing [Cl*^–^*]_*i*_ in *Cftr*^–/^*^–^* enterocytes (Walker et al., 2016). Employing an array of sophisticated methods, the group demonstrated an expression and functional activity of CFTR in murine ISCs, an alkaline intracellular pH_i_ in *Cftr*^–/^*^–^* ISCs, accompanied by hyperproliferation in *Cftr*^–/^*^–^* organoids. These findings suggest that the *Cftr*^–/^*^–^*-associated crypt and villus elongations, which are also observed in the absence of inflammatory markers (Tan et al., 2020) and the hyperproliferation described in murine *Cftr*^–/^*^–^* intestinal epithelium (Gallagher and Gottlieb, 2001) may be partially a consequence of the lack of CFTR in ISCs. Crossbreeding of *Cftr*^–/^*^–^* and *wt* mice with transgenic mice which express a fluorophore (EGFP)-labeled WNT transducer Disheveled (Dsv) and the cell membrane-targeted, two-color fluorescent Cre-reporter Rosa^TmT/mG^ enabled the group to study the proximity of Dsv to the membrane receptor Frizzled 7, which has been recognized as a key event in WNT signaling (Axelrod, 2001) in *Cftr*^–/–^ and *wt* ISCs with live cell imaging. Subjecting the organoids to manipulations that reduced inner membrane negative charge, [Cl]_i_ or pH_i_, the authors established the causative role of pH_i_ alkalinity for increased WNT signaling in *Cftr*^–/–^ ISCs.

Loss of CFTR function in CF patients is associated with a significantly increased risk of developing digestive tract cancers, but not of lung cancers (Neglia et al., 1995; Maisonneuve et al., 2003, 2013; Scott et al., 2020). CFTR is expressed in ISCs, but not detectable in the progenitor basal cells of the respiratory mucosa (Plasschaert et al., 2018). Since both organs are subjected to the typical CF epithelial manifestations of dysbiosis, inflammation, and remodeling, the findings by Strubberg et al. (2018) may have identified an intrinsic factor favoring malignant growth in the CF intestinal epithelium.

### Associations Between pH_i_ and/or Acid/Base Transporters and Epithelial Morphogenesis in Native Intestinal Epithelium

From the existing literature pool, only the study done by Strubberg et al. (2018) provides a molecular mechanism linking steady-state pH_i_ and proliferation in native epithelium. In a number of cellular systems, however, it is found that slightly alkaline pH (∼0.3 pH units above the steady-state pH_i_) is important for initiating DNA synthesis and proliferation [reviewed in Flinck et al. (2018)]. Here we report studies in native intestinal epithelium in which pH_i_ and/or proliferation was measured, but the molecular mechanism linking the two has yet to be explored.

SLC4A4 (NBCe1) is expressed predominantly in small intestinal villous (Jakab et al., 2011) and colonic surface cells and NBCe1 KO proximal surface colonocytes have significantly reduced steady-state pH_i_ compared to WT (Yu et al., 2016). Due to the short life span and the tiny intestine of these mice, the proliferation rate in the colon was not addressed, but a study with LS174 cells (human colonic adenocarcinoma cells) showed that SLC4A4 knockdown impaired cell proliferation (Parks and Pouyssegur, 2015). The Cl^–^/HCO_3_^–^ exchanger Slc26a3, which mutation is the molecular cause of congenital chloride diarrhea, is mainly expressed in the colonic absorptive epithelial cells lining the luminal surface, not in the ISCs (Hoglund et al., 1996; Xiao et al., 2012). Loss of Slc26a3 function, in the colon leads to increased steady state pH_i_ in the surface epithelium (Xiao et al., 2014). Colonic epithelial hyperplasia has been described in the original publication of the *Slc26a3*^–/–^ mouse (Schweinfest et al., 2006). Possibly the alkaline pH interferes with intracellular acidification required to trigger programmed cell death at the colonic surface epithelium (Park et al., 1999; Kniep et al., 2006). Increased crypt length, seen in *Cftr*^–/–^ epithelium, is not a feature of *Slc26a3*^–/–^ colonic epithelium (Kini et al., 2020).

What about the effect of an acidic pH_i_ on proliferation and differentiation? While many cell lines are viable in acidic medium, the pharmacological or genetic inhibition of acid extruders or base loaders generally curbs proliferation and has been repeatedly suggested as an antiproliferative treatment in tumors (Chambard and Pouyssegur, 1986). It was recently reported that a genetic deletion of an acid extruder, namely NHE8, in colonic ISC displays hyperproliferative phenotype, but the pH_i_ in the affected cells was not measured (Xu et al., 2019).

### Cellular Signaling Pathways That May Link pH_i_ to Proliferation and Differentiation

Regeneration, expansion and lineage differentiation of the intestinal epithelium is modulated by various signaling pathways, namely Wnt/β-catenin, EGF (epidermal growth factor), BMP (bone morphogenetic protein), Notch, Hedgehog, and Eph–ephrin which mainly occur in gradients along the crypt/villus axis as depicted in Figure 1 (Spit et al., 2018). These signaling cascades are derived from the epithelial or the mesenchymal niche (Spit et al., 2018). A potential cross-interaction between the gradients of signaling pathways and the intracellular pH gradient along the crypt/villus axis might exist, but is understudied. In this paragraph we point out important signaling pathways for proliferation and differentiation in which a relationship to pH_i_ has been delineated in other cellular system, and which are worth studying in the native intestinal epithelium.

Wnt signaling is the main driving force of ISC proliferation. Increased Wnt/β-catenin signaling leads to hyperproliferation observed in *Cftr*^–/–^ ISCs as described above. The molecular mechanism behind the increased Wnt activity involves the alkaline pH_i_-facilitated association of the Wnt transducer Disheveled (Dvl) to the plasma membrane and binding to the Frizzled-7 receptor (Fz) (Walker et al., 2016; Strubberg et al., 2018). Similarly, a study in *Drosophila melanogaster* cells shows that the activity of dNhe2 (a *Drosophila* analog of the mammalian NHE1), which allows maintenance of alkaline pH_i_, is necessary for the binding and surface recruitment of Dvl by Fz (Simons et al., 2009). Another component of the Wnt signaling pathway, β-catenin is also influenced by the pH_i_. Increasing the pH_i_ by glycolysis stimulates β-catenin acetylation leading to Wnt signaling activation in embryos and human tail bud-like cells differentiated *in vitro* from iPS cells (Oginuma et al., 2020). Intracellular acidification induces the transcriptional repressor DDIT3 that suppresses the activity of Wnt, as shown *in vitro* and in a mouse xenograft tumor model (Melnik et al., 2018). However, another group has shown that cell alkalization with NH_4_Cl in MDCK epithelial cells and *Drosophila melanogaster* led to decreased β-catenin abundance at cell–cell junctions and in the nucleus (White et al., 2018), while lower pH_i_ in NHE1-deficient PS120 fibroblasts significantly increased β-catenin at membrane protrusions (White et al., 2018). Recently the involvement of potassium channels, namely KCNQ1, in the Wnt/β-catenin signaling pathway has been shown. A colocalization of KCNQ1and β-catenin at the adherence junctions was detected in rat colonic crypts and HT29 cells (Rapetti-Mauss et al., 2017) and KCNQ1 inhibition leads to re-localization of β-catenin in the cytosol, Wnt/β-catenin signaling pathway stimulation with increased expression of Cyclin D1 and C-Jun as Wnt target genes (Rapetti-Mauss et al., 2017, 2020). Although, pH_i_ is not directly implied, K^+^ channels allow hyperpolarization of the membrane voltage, thus contributing indirectly to pH regulation (Spitzner et al., 2007).

EGF signaling is another important modulator of ISC proliferation. EGF is produced by the adjacent fibroblasts and Paneth cells (Sato et al., 2011; Farin et al., 2014), and activates the signaling cascade by binding to the EGF receptor (EGFR) on ISCs. Extracellular pH influences the binding of EGF to its receptor, with maximized binding at pH8 and reduced interaction at pH6.5 (Nunez et al., 1993). Early research showed that EGF can increase pH_i_ in A431 human epidermoid carcinoma cell line (Rothenberg et al., 1983). Later investigation in chicken granulosa cells (Li et al., 1991), Hep G2 hepatoma cells (Strazzabosco et al., 1995), and in primary cultured rabbit surface epithelial cells (Nylander-Koski et al., 2006) showed that EGF induced intracellular alkalization occurs via activation of NHE1. These data point that an alkaline pH_i_ shift, caused by activation of NHE1 on the basolateral membrane, is an important event in EGF signaling pathway that stimulates cell proliferation. Indeed, EGFR forms a complex with NHE1 via NHERF1 (Cardone et al., 2015). Apical EGF can also activate EGFR signaling and promote proliferation similarly to basolateraly induced EGFR activation (Kuwada et al., 1998). EGF impacts the apical NHEs, it stimulates NHE2 mRNA expression and activity in rat intestinal epithelial (RIE) cell (Xu et al., 2001), but it has a negative effect on NHE8 expression (Xu et al., 2010). The interplay between apical or basolateral EGFR activation, different NHEs, and the pH_i_ is not completely understood. It seems plausible that a constant slightly acidic pH_i_ in the ISC zone may prevent hyperactivation of the signaling pathways that could result in hyperproliferation and possible tumor formation (Liu et al., 2020).

BMP, Notch, Hh, and Eph are more dominant in the upper sections of the crypt and involved in cell fate decision and terminal differentiation. Knowledge about the impact of the pH_i_ on the later signaling cascades is scarce. The activation of the Notch signaling involves binding of the ligands to the receptor, and subsequent activation of the endocytosis machinery and this later step is influenced by the vacuolar (H^+^)-ATPase (V-ATPase), a proton transporter involved in the acidification of endosomal compartments (Yan et al., 2009). Notch signaling plays an important role in the determination of cell fate by regulating the balance between cell proliferation and differentiation (Baron, 2003), thus impacting the transit amplifying cells. Therefore, there is a high possibility that the Notch signaling can be affected by the activity of ion transporters present in the transit amplifying cells, such as NHE2 (Guan et al., 2006) via fine-tuning the pH_i_ value. A shift in the pH_i_ toward more alkaline values (from 7.4 to a 7.65) has been observed in mouse embryonic stem cells during their differentiation *in vitro* (Ulmschneider et al., 2016). Hh signaling, important for the follicle stem cells differentiation in *Drosophila*, is also strongly influenced by pH_i_ alterations.

The reported studies are only examples, which, taken together, suggest that the pH_i_ may trigger stimulation or inhibition of different signaling pathways active in proliferation and differentiation of the intestinal epithelium. However, the exact molecular mechanism correlating the pH_i_ and signaling pathways gradient in the intestinal epithelium is yet to be determined.

## Conclusion and Outlook

Addressing the role of the steady-state pH_i_ in intestinal epithelial homeostasis has been hampered by the absence of models that accurately assess the pH_i_ in different epithelial compartments along the crypt-villus axis, and to induce long-term and selective pH_i_-alterations. The ability to generate intestinal organoids and monitor their growth over days in culture has enabled scientists to intensely study the process of intestinal cell renewal and differentiation. Some of the molecular events that link the elevated pH_i_ secondary to loss of functional CFTR to intestinal epithelial hyperproliferation were elegantly explored by generating intestinal organoids from *Cftr^–/–^* and *wt* mice crossed onto a variety of transgenic reporter mouse lines (Strubberg et al., 2018). These seminal studies may provide clarification of the increased incidence of colorectal cancer in CF patients and contribute toward their prevention. Recent progress in the drug development for CFTR corrector and modifiers with the potential to rescue CFTR function in CF patients may correct the pH_i_-regulatory dysfunction and reduce cancer risk (Phuan et al., 2019; Egan, 2020). The potential to combine direct pH_i_ assessment with genetic, molecular biological and pharmacological tools, as already established for tumor cells (Liu et al., 2020; Stock, 2020), in intestinal organoids may provide insight into protonation/deprotonation events of key regulatory proteins in enterocyte proliferation and differentiation.

## Author Contributions

MA, US, and KN designed and wrote the review. All the authors contributed to the article and approved the submitted version.

## Conflict of Interest

The authors declare that the research was conducted in the absence of any commercial or financial relationships that could be construed as a potential conflict of interest.

## Abbreviations

ISCs: intestinal stem cells
CFTR: cystic fibrosis transmembrane conductance regulator
SLC26A3: Solute Carrier Family 26 Member 3 (Cl^–^/HCO_3_^–^ exchangers)
NHE1/2/3/8 (SLC9A): sodium/hydrogen exchangers 1/2/3/8
AE2 (SLC4A2): Anion exchange protein 2
NBCn1 (SLC4A7): Electroneutral sodium/bicarbonate-dependent cotransporter
NBCn1 (SLC4A4): Electrogenic sodium/bicarbonate cotransporter
GI: gastrointestinal
WT: wild type
KO: knock out
CF: cystic fibrosis
EGF: epidermal growth factor
BMP: bone morphogenetic protein
Dvl: Disheveled
Fz: Frizzled receptor
Hh: Hedgehog signaling.
